# Simulation Study of Xylitol-Mediated Effect on NaCl Diffusion Behavior in Cured Pork Tenderloin

**DOI:** 10.3390/foods12071451

**Published:** 2023-03-29

**Authors:** Dan Chen, Qiujin Zhu, Ying Zhou, Jing Wan, Li Deng, Lei Wang, Linggao Liu, Sha Gu, Yanpei Huang, Yeling Zhou, Shenghui Bi

**Affiliations:** 1 School of Liquor and Food Engineering, Guizhou University, Guiyang 550025, China; 2 Key Laboratory of Agricultural and Animal Products Store and Processing of Guizhou Province, Guiyang 550025, China; 3 Key Laboratory Mountain Plateau Animals Genetics and Breeding, Ministry of Education, Guiyang 550025, China

**Keywords:** diffusion coefficient, cured meat, xylitol, simulation, COMSOL multiphysic

## Abstract

Polyhydroxy alcohol-mediated curing has great potential for producing low-salt cured meat products. This study investigated the mass transfer kinetics and the one-way diffusion simulation of sodium chloride (NaCl) during the curing process. Furthermore, Fick’s second law determined the NaCl diffusion coefficient (De) of xylitol-mediated cured pork tenderloin. The results demonstrated that adding xylitol could reduce the De of NaCl. The De of NaCl, calculated using the one-way model, was 1.29 × 10^−9^ m^2^·s^−1^, 1.22 × 10^−9^ m^2^·s^−1^, 1.2 × 10^−9^ m^2^·s^−1^, and 1.15 × 10^−9^ m^2^·s^−1^ when the amount of xylitol added was 0%, 4%, 8%, and 12% (*w*/*w*), respectively. This result agrees with the predicted values from the power function time-varying model. Moreover, a three-dimensional simulating model of mass transfers constructed using COMSOL Multiphysics was developed to evaluate the NaCl diffusion in pork tenderloin during the curing process. This model has high accuracy and can be used to describe the diffusion of NaCl in curing. Overall, this study provided a foundation for NaCl diffusion and distribution during the curing process.

## 1. Introduction

The curing process has been applied for several years and is an effective tool to preserve meat, playing an essential role in improving the flavor and color of meat [1]. Currently, some reports focus on improving the curing efficiency, low sodium salt, and kinetic changes [2,3]. Numerous studies have shown that excessive daily salt intake leads to high blood pressure, stroke, stomach cancer, and related non-communicable diseases [4,5]. Current methods to reduce salt content in cured meat products can be divided into three categories [6]: changes to the physical form of salt or use of alternative salt [7,8]; use of a new curing and processing technology or improving the curing and processing technology [9]; or change in the diffusion rate of sodium chloride (NaCl) [10,11]. Therefore, this study aimed to propose a new curing strategy, mediated curing. The method of curing by adding exogenous substances without changing the properties of the salt itself is called exogenous substance-mediated curing [12]. The studies performed until now are limited to the effects of different exogenous compounds on the product’s food quality and shelf life [13]. However, the mechanism of the effect of these compounds against the mass transfer rate of NaCl in curing meat is still unclear. Xylitol has three hydroxyl groups in its molecular structure, and these hydroxyl groups are able to bond with hydroxyl groups of water in the form of hydrogen bonds. Therefore, adding xylitol can extend the shelf life of meat products and improve the water-holding capacity of meat products. Xylitol is one of the most widely used polyhydroxy sugars, with good thermal stability and moisture absorption. Xylitol has a good market prospect in the food industry and is considered to be the best substitute for sucrose [14].

The research on mass transfer can be divided into macroscopic and microscopic perspectives. For example, studying diffusion from a microscopic perspective usually requires large and precise instruments with relatively high costs [15,16,17]. Comparatively speaking, computer modeling could be an economical and fast method to describe the spatial distribution of water and salt in several products. Currently, software, including FLUENT, CFX, MATLAB, and COMSOL Multiphysics [18,19], can be applied in the food industry. For example, Shi et al. [20] performed 3D and 2D simulations of the diffusion behavior of the marinade during the beef marination process using COMSOL Multiphysics to predict the transient distribution state of the marinade. Until now, there are no relevant reports on the computer modeling of exogenous-mediated substances with respect to the mass transfer of NaCl during the meat-curing process.

Therefore, this study determined the effect of xylitol on the NaCl mass transfer rate of pork tenderloin. The diffusion of NaCl in the curing pork tenderloin process using xylitol was simulated using COMSOL Multiphysics software (COMSOL Multiphysics 5.5) and R language (R Core Team, Vienna, Austria, 2019). This study could be helpful to visualize the diffusion and distribution of NaCl in the process of curing pork tenderloin.

## 2. Materials and Methods

### 2.1. Materials

In this study, pork tenderloin was obtained 48 h postmortem from a slaughterhouse of Fuzhiyuan Food Co., Ltd. (Guiyang, China). The NaCl was purchased from Biyuan Salt Co., Ltd. Guiyang, China. Food-grade xylitol was purchased from Huakang Pharmaceutical Co., Ltd. (Quzhou, China). Other chemicals were of analytical grade and purchased from Sinopharm Chemical Reagents Co., Ltd. (Shanghai, China).

### 2.2. Methods

#### 2.2.1. Cured Pork Tenderloin Sample Preparation

Three pigs were selected at random from the same slaughterhouse. Samples were taken from pork loins (Longissimus dorsi) 10–20 cm from the hip of three animals at approximately 48 h post-mortem. The visible fat and connective tissue were removed from the surface of the fresh pork tenderloin and cut into flat samples (30 × 30 × 10 [length ×width × height] mm). Then, the mass of all samples used in this study was defined as 10 ± 1 g. A total of 618 samples were used, and the 618 samples were divided into three parts; 576 samples were used for the measurement of mass transfer kinetic parameters at different xylitol concentrations during loin curing, 36 samples were used for the determination of the diffusion coefficient of NaCl in the unidirectional model and the determination of the model, and 6 samples were used to verify the validity of the visualization model [21]. Then, the samples were packed under a vacuum and stored at −80 °C for further use. Then, the samples were randomly selected and thawed at 4 °C for 24 h for curing. After, these samples were immersed in beakers containing 8% (*w*/*w*) NaCl solution with 0%, 4%, 8%, 12% (*w*/*w*) xylitol addition, respectively, at 4 °C without stirring, and the weight ratio of pork tenderloin to the salt solution was 1:4 (*w*/*w*). Then, the samples were collected every 2 h and immersed in distilled water for 20 s to remove the excess salt. Finally, the water on samples surface was dried with absorbent paper, weighed, and preserved at 4 ± 1 °C for further analysis.

#### 2.2.2. Measurements of Moisture and NaCl Contents

In this study, 3 g of the samples was used to determine the moisture content at 24 ± 1 °C using a meat moisture analyzer (SFY-30, Guanya, Shenzhen, China). Additionally, the NaCl content was determined in triplicate using a digital salt meter (ES-421, ATAGO, Tokyo, Japan) according to a previous method [22].

#### 2.2.3. Evaluation of Changes in Water, Salt, and Total Weight

The total weight change amount (Δ$M_{t}^{0}$), moisture change amount (Δ$M_{t}^{w}$), and salt change amount (Δ$M_{t}^{NaCl}$) of the pork tenderloin after the curing process were calculated using the following Equations (1)–(3) [23]:

(1) $$\Delta M_{t}^{0}\%=\frac{M_{t}^{0}-M_{0}^{0}}{M_{0}^{0}}\times 100\;$$

(2) $$\Delta M_{t}^{w}\%=\frac{M_{t}^{0}\times X_{t}^{w}-M_{0}^{0}\times X_{0}^{w}}{M_{0}^{0}}\times 100\;$$

(3) $$\Delta M_{t}^{NaCl}\%=\frac{M_{t}^{0}\times X_{t}^{NaCl}-M_{0}^{0}\times X_{0}^{NaCl}}{M_{0}^{0}}\times 100\;$$

where $M_{t}^{0}$ and $M_{0}^{0}$ represent the weights of samples (g); $X_{t}^{w}$ and $X_{0}^{w}$ represent the moisture content; and $X_{t}^{NaCl}$ and $X_{0}^{NaCl}$ represent the salt content at the corresponding curing times t (h) and 0 (h), respectively.

#### 2.2.4. Construction of Mass Transfer Kinetic Model

The kinetic model of mass transfer by curing was constructed using Equation (4) [24]:

(4) $$\Delta M_{t}^{i}=1+k_{1}+k_{2}\times t^{0.5}\;$$

where $\;\Delta M_{t}^{i}$ includes the $\Delta M_{t}^{0}$, $\Delta M_{t}^{w}$, and $\Delta M_{t}^{NaCl}$ during the curing process; the intercept *k*_1_ was used to describe the parameters at the initial stage of curing. The slope *k*_2_ was associated with the mass transfer kinetics of the diffusion mechanism of mass transfer kinetics about the curing.

#### 2.2.5. NaCl Diffusion Coefficient (De)

The NaCl content in the aqueous phase of the pork tenderloin ($Z^{NaCl}$) was determined by Equation (5).

(5) $$Z^{NaCl}=\frac{X^{NaCl}}{X^{w}+X^{NaCl}}\;$$

When the curing process reached the equilibrium phase, the NaCl content in the aqueous phase of the loin ($Z_{e}^{NaCl}$) was equal to the content in the curing solution ($Y_{e}^{NaCl}$), as described in Equation (6) [25]:

(6) $$Z_{e}^{NaCl}=Y_{e}^{NaCl}=\frac{\frac{M_{0}^{SD}}{M_{0}^{SS}}\times X_{0}^{S}+y_{0}^{s}}{\frac{M_{0}^{SD}}{M_{0}^{SS}}\times \left(X_{0}^{w}+X_{0}^{s}\right)+\left(y_{0}^{w}+y_{0}^{s}\right)}\;$$

where $M_{0}^{SS}$ is the initial mass of the pork tenderloin (g); $M_{0}^{SD}$ is the initial mass of brine solution (g); $X_{0}^{s}$ is the salt content of the pork tenderloin at the beginning of the curing process (%); $X_{0}^{w}$ is the moisture content of the pork tenderloin at the beginning of the curing process (%);$\;y_{0}^{s}$ is the salt content of the brine solution at the beginning of the curing process (%); and $y_{0}^{w}$ is the moisture content of the brine solution at the beginning of the curing process.

It has been described that, according to Fick’s second law, the samples can be considered as an isothermal semi-infinite geometry slab with unidirectional mass transfer. Furthermore, in this study, the NaCl diffusion coefficient was assumed as a constant and determined using Equation (7) [26]:

(7) $$1-Y_{t}^{NaCl}=1-\left[\frac{\left(z_{t}^{NaCl}-y_{t}^{NaCl}\right)}{\left(z_{0}^{NaCl}-z_{e}^{NaCl}\right)}\right]=2\times {\left(\frac{De\times t}{\pi \times L^{2}}\right)}^{0.5}+K\;$$

where $Y_{t}^{NaCl}$ is the driving force between the aqueous phase of the pork tenderloin and brine (%); $y_{t}^{NaCl}$ is the NaCl content in the brine (%); t is the curing time (h); L is the thickness of the sample (m); *K* is a constant; and $z_{t}^{NaCl}$, $z_{0}^{NaCl}$, and $z_{e}^{NaCl}$ represent the NaCl content in the aqueous phase of pork tenderloin at different curing times (t, 0 and e [equilibrium]), respectively (%).

#### 2.2.6. Evaluation of the Link between NaCl Content and Diffusion Distance

To determine the link between NaCl content and diffusion distance in cured pork tenderloin, the samples were collected at the end of the curing process, rinsed with distilled water for 20 s, and dried with absorbent paper. After, the meat was frozen in liquid nitrogen. Then, the samples were sliced into 5 slices (2 mm), and the NaCl and water contents were separately measured.

### 2.3. One-Way Model Simulations of the Kinetics of NaCl Diffusion

Samples were embedded in paraffin, except for one flat surface perpendicular to the direction of pork tenderloin muscle fibers. After the curing process, the paraffin was removed from the surface, rinsed with distilled water for 20 s, and dried with absorbent paper for further analysis [27].

In this assay, two groups were created. In one group, the samples were removed at 2 h intervals to establish a predictive model of the NaCl content in the aqueous phase of the tenderloin over time and to calculate the diffusion coefficient of NaCl. On the other hand, the second group was used to verify the validity of the simulating model for predicting the NaCl diffusion in pork tenderloin during the curing. Therefore, the samples were collected in this group after 12 and 24 h. Afterwards, the samples were freeze-dried under a vacuum (cold trap temperature −80 °C, and vacuum degree 1.9 Pa) and cut into slices of 2 mm running orthogonally to the NaCl transfer direction (the actual thickness was calculated as a percentage of the total weight by weight). The NaCl content of each slice sample was measured. 

This study used the linear time-variant model, power function time-variant model, and exponential time-variant model to evaluate the relationship between NaCl content and time in the aqueous phase of the pork tenderloin [28].

Linear time-variant model:

(8) $$Z^{NaCl}=At+b\;$$

Power function time-variant model:

(9) $$Z^{NaCl}=At^{b}+c\;$$

Exponential time-variant model:

(10) $$Z^{NaCl}=A1-e^{bt}\;$$

where *A*, *b*, and *c* are constants. $Z^{NaCl}$ is the NaCl content in the aqueous phase of pork tenderloin, calculated using Equation (5).

Considering the sample as an isothermal and semi-infinite material with unidirectional mass transfer and a constant diffusion coefficient along the *x*-axis, the De of NaCl was calculated using the following formula. Since in this study, only one flat surface of the meat sample was exposed to the saline and saline did not enter the sides of the vessel, this model was applied to the experimental setup as previously described [17].

(11) $$\frac{Cs-C\left(x,\;t\right)}{Cs-C_{0}}=erf\left(\frac{x}{2\sqrt{De\Delta t}}\right)\;$$

where Cs is the concentration of NaCl in the brine (%); C(x, t) is the NaCl content in the pork tenderloin at a distance of x along the myogenic fiber direction at moment *t* (%); $C_{0}$ is the initial NaCl content (%) in the pork tenderloin; *x* is the distance along the direction of myogenic fibers (m); *De* is the effective diffusion coefficient; *t* is the curing time (h); and the erf is the function, calculated using R language.

### 2.4. Statistical Analysis

Statistical analysis was performed using the SPSS software (version 22.0, SPSS Inc., Chicago, IL, USA). The content of moisture and NaCl was analyzed using ANOVA, and the significant differences (*p* < 0.05) between the individual means were identified with least significant difference multiple comparisons, and relevant experimental results are reported as the mean ± S.E.

The linear time-variant model (Equation (8)), power function time-variant model (Equation (9)), and exponential time-variant model (Equation (10)) were used for the NaCl content prediction. In the preliminary experiment, it was found that the treatment groups showed no significance between each other when the xylitol concentration was increased from 4% to 12% (*w*/*w*). Therefore, the diffusing simulation was performed for curing tenderloin treated with 4% (*w*/*w*) xylitol. Moreover, three representative locations on the same piece of curing meat were chosen to determine the moisture content, thus ensuring that all samples possessed a constant concentration of 8% (*w*/*w*) NaCl.

## 3. Results and Discussion

### 3.1. Total Weight, Moisture, and NaCl Contents

**Figure 1:**
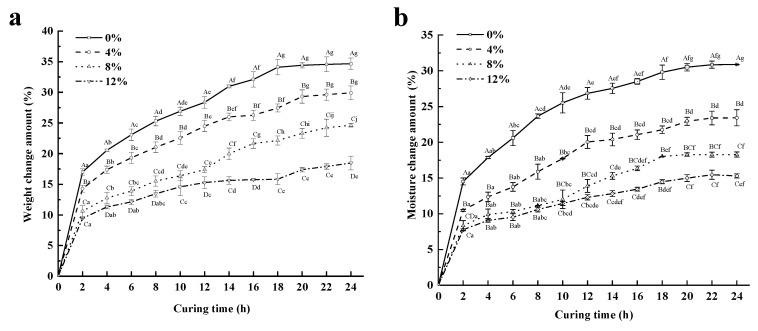

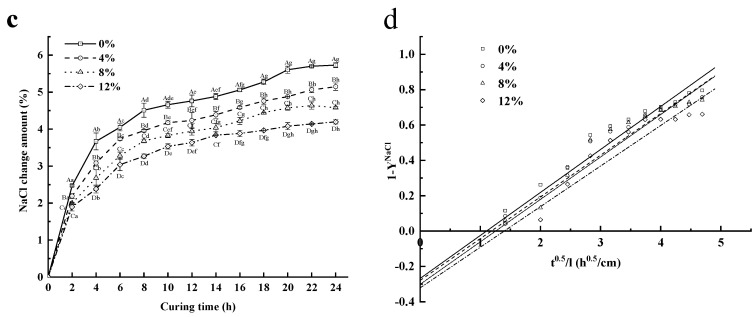
The weight change amount (**a**), moisture change amount (**b**), and NaCl change amount (**c**) of pork tenderloin with different xylitol concentrations, and driving force (1 − Y_t_^NaCl^) versus curing time (t^0.5^/l) (**d**). Note: different capital letters indicate significant differences between the treatment groups with different concentrations of xylitol (*p* < 0.05); different small letters indicate significant differences between the treatment groups with different curing processes (*p* < 0.05).

The results demonstrated that xylitol had significant effects (*p <* 0.05) on the weight, moisture, and NaCl contents of pork tenderloin during curing (Figure 1). Furthermore, the addition of xylitol induced a weight change greater than 0, and the production rate was increased (Figure 1a). Xylitol is adsorbed on the surface of the tenderloin, forming a “barrier effect” that prevents NaCl entrance into the tenderloin. Furthermore, this compound contains several hydroxyl groups, which bind to some proteins in meat, participating in protein reactions. This binding can induce microstructural changes in the muscle, promoting the conversion of free water to bound water and less mobile water in the muscle, reducing the ability of water to penetrate the muscle tissue. Therefore, adding xylitol can reduce the weight change amount of the tenderloin.

Initially, the NaCl in the brine entered the superficial muscle forming an osmotic system with the muscle cells, water kept leaking out and the solute infiltrated, and the amount of water change tended to progress towards stability after 20 h. Furthermore, an increase was detected in the moisture content in the meat during the curing process (Figure 1b). However, no significant differences were detected in the moisture change amount of the tenderloin (*p* > 0.05) when the xylitol concentration was higher than 8% (*w*/*w*). This result could be explained because xylitol can gradually penetrate the tissue cells until saturation, and the influence of the water content on the tenderloin due to the xylitol concentration begins to shrink [29].

The results in Figure 1c showed that the NaCl content of tenderloin increased faster in the first 6 h of curing, and stabilization was observed after 20 h. The material transfer during the curing process can be characterized by dynamic and equilibrium periods [30]. In the initial stage, the osmotic pressure of the brine solution outside was higher than that in the muscle cells; therefore, NaCl diffusion was at a high rate. The difference in NaCl content between the experimental and control groups (none) is evidence that adding xylitol hinders the diffusion of NaCl in the meat, thus reducing the salt content in meat products. In the late stage of curing, the amount of NaCl changed in all samples and showed a slow increase because the osmotic pressures of the marinade and the meat were gradually equalized, and the material transfer inside and outside the tissue cells reached equilibrium. In addition, during muscle water absorption, the moisture change amount (Figure 1b) was always higher than the NaCl change amount (Figure 1c) because the mass transfer resistance of Na^+^ and Cl^−^ was higher than that in water molecules [22].

### 3.2. Application of Predictive Models to Detect Changes

**Figure 2:**
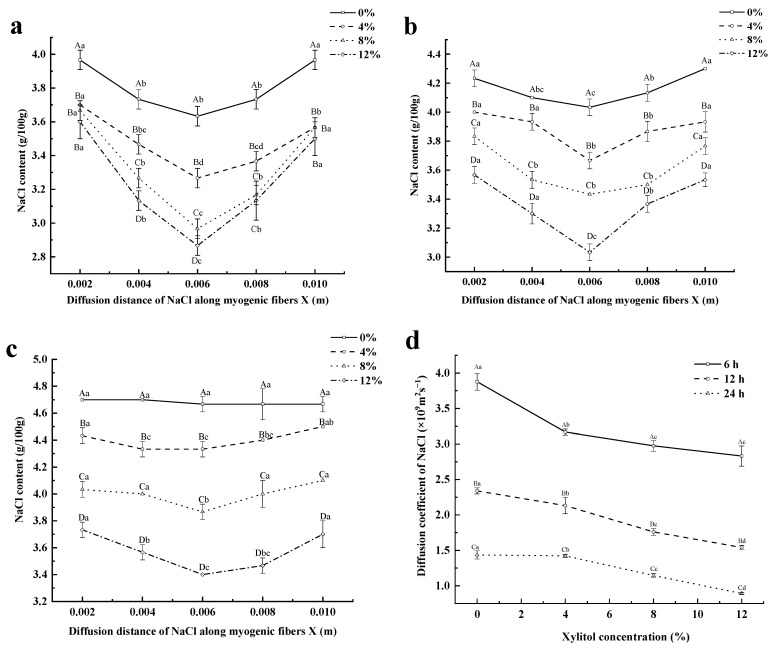
Variation in NaCl content with diffusion distance in pork tenderloin cured for 6 h (**a**), 12 h (**b**), and 24 h (**c**) based on different concentrations of xylitol, and the effect of the xylitol concentration and curing time on the diffusion coefficient De of NaCl (**d**) (×10^−9^ m^2^·s^−1^). Note: different capital letters indicate significant differences between the treatment groups with different concentrations of xylitol (**a**–**c**), and curing times (**d**) (*p* < 0.05); different small letters indicate significant differences between the treatment groups with different diffusion distances (**a**–**c**) and different concentrations of xylitol (**d**) (*p* < 0.05).

R language version 4.1.0 was used to calculate the De values of NaCl for different concentrations of xylitol-mediated cured pork tenderloin, the curve of loin weight variation during mediated curing using the prediction model in Figure 2, and the parameters of the regression equation. Therefore, using this software, the NaCl diffusion coefficients were obtained (Table 1a). The diffusion coefficient of NaCl became slightly smaller when xylitol was added, ranging from 2.35 to 2.58 × 10^−9^ m^2^·s^−1^. This result indicates that xylitol slightly affected the diffusion coefficient of NaCl. Moreover, the De values obtained in this study were smaller than experimental results previously described [31]. This may be related to several parameters, such as the material–liquid ratio, NaCl content of the brine, xylitol concentration, and curing temperature [32].

The variation in total weight ($\Delta M_{t}^{0}$), moisture content ($\Delta M_{t}^{w}$), NaCl content ($\Delta M_{t}^{NaCl}$), and the parameters k_1_ and k_2_ are shown in Table 1b. The intercept parameter K_1_ is used to describe the initial stage of curing, which is mainly affected by the salt concentration gradient, water gradient, pressure gradient, and other factors during the initial stage of curing. The slope parameter K_2_ is related to diffusion kinetics and is affected by the type of additive [33]. The changes in weight, moisture content, and NaCl content of tenderloin had good linear relationships (R^2^ ≥ 0.9000) with curing time (t^0.5^) under different xylitol concentrations. These results suggest that the model can be applied to simulate xylitol-mediated mass transfer during the curing process. Table 1b shows that the concentration of xylitol has a significant impact on K_2_; this is because the xylitol-mediated curing can significantly reduce the mass, moisture content, and NaCl content. However, the addition of xylitol had no significant impact on the NaCl concentration under the initial curing conditions, so the change in K_1_ was not significant.

### 3.3. Link between NaCl Content and Diffusion Distance

In this study, the link between the NaCl content in tenderloin and diffusion distance at 6 h (Figure 2a), 12 h (Figure 2b), and 24 h (Figure 2c) of xylitol-mediated curing of pork tenderloin was evaluated. After 6 and 12 h, the NaCl content exhibited a decrease. However, an increase was detected when the diffusion distance was enhanced. In addition, the NaCl content in the slices of meat at a 2 mm thickness was very close to that in the whole cured tenderloin, which was due to the high surface-NaCl content, and the penetration of NaCl was mainly concentrated on the surface of the meat [34]. Therefore, the results indicate that when the distance of the meat was increased, a decrease in the NaCl content was observed.

Because xylitol adsorbed on the surface of the loin during the curing, forming a “barrier effect” that prevented the loin from absorbing NaCl and effectively reduced the salt content [35], the group treated with xylitol-mediated curing showed significantly reduced NaCl content on the surface and inside the meat products (*p* < 0.05) (Figure 2a). No significant differences were detected in the surface NaCl concentrations among the different groups treated with different xylitol concentrations after 6 h (*p* > 0.05). This may be because at the early stage of curing, most of the NaCl remained on the surface of the loin, the amount of NaCl diffused into the interior was still small, and the difference in surface NaCl content between the 4–12% (*w*/*w*) xylitol treatment groups was not significant (*p* > 0.05).

The results in Figure 2b demonstrated that the concentration of NaCl on the surface of the experimental group was significantly lower than that in the control group in the middle of the curing process (*p* < 0.05). Furthermore, after 24 h of curing, the group treated with xylitol reached equilibrium, and NaCl diffused evenly into the loin (Figure 2c). Therefore, no significant differences were observed in the NaCl content when the diffusion distance was increased (*p* > 0.05). On the other hand, the results also showed that the average salt content of the meat block was 4.7%. During the same curing time, the salt content of meat products in the xylitol group was significantly lower than that in the control group (*p* < 0.05). This result suggests that adding xylitol had a specific hindering effect on the diffusion of NaCl during the curing process.

The mass transfer flux in the curing process is mainly the change in the transport of water-soluble compounds (mainly NaCl) or water itself in the sample water phase. Therefore, NaCl content in the water phase of the tenderloin is used instead of NaCl content in the tenderloin [36]. A decrease was observed in the De values with the curing time in all groups (Figure 2d). This result agrees with a previous study [37]. The infiltration of NaCl during curing leads to myosin dissolution and changes in the structure of the meat matrix, and the muscle extracellular space may become more viscous, thereby increasing mass transfer resistance. In addition, De decreased significantly with the xylitol concentration (*p* < 0.05), which may be related to the hydroxyl groups in xylitol that bind to protein molecules in the meat and participate in protein reactions [38]. This binding can induce microstructural changes in the muscle, promoting the conversion of free water to bound and less mobile water in the muscle, decreasing the osmotic pressure difference between the meat and the brine solution, and altering the ability of NaCl to penetrate the muscle tissue [11].

### 3.4. Simulating Model

**Figure 3:**
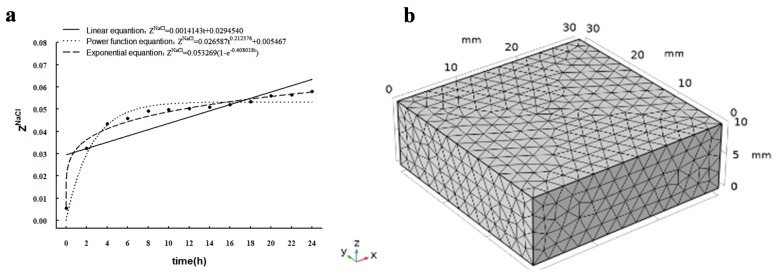
Fitted equations of time and NaCl content with 4% xylitol in pork tenderloin (**a**) and 3D grid model of pork tenderloin (**b**).

In this study, the linear function time-varying, power function time-varying, and exponential function time-varying equations were used to predict the NaCl content in the aqueous phase of the pork tenderloin during the curing process (Figure 3a). R^2^ and De under these prediction models are shown in Table 2. The R^2^ cases for the relationship between the NaCl content in the aqueous phase of the tenderloin and the curing time can be obtained as follows: power function time-varying model > exponential function time-varying model > linear function time-varying model. The power and exponential functions (R^2^ > 0.9500) are good for correlations when the linear function is poor (R^2^ < 0.7). The results obtained in this study agree with previous studies [39], which proves that the data predicted by the power function time-varying model are closer to the De value calculated using the experimental method, and the experimental data were a good fit with predicted values. Additionally, when xylitol was added (0–12%), the experimentally calculated De values were 1.29 × 10^−9^ m^2^·s^−1^, 1.22 × 10^−9^ m^2^·s^−1^, 1.2 × 10^−9^ m^2^·s^−1^, and 1.15 × 10^−9^ m^2^·s^−1^ (Figure 2d). The power function time-varying model was 1.29 × 10^−9^ m^2^·s^−1^, 1.21 × 10^−9^ m^2^·s^−1^, 1.14 × 10^−9^ m^2^·s^−1^, and 1.04 × 10^−9^ m^2^·s^−1^ (Table 2). However, no significant differences were observed between the experimental conditions (*p* > 0.05). Andrés et al. showed that the change in mass transfer during curing was related to the square root of time. The square root time-varying model is a subset of the power function time-varying model, so the power function time-varying model is used to predict the NaCl content in the water phase of the pig ridge at different treatment times and calculate the diffusion coefficient of NaCl [40]. Therefore, the power function time-varying model was subsequently chosen as the prediction equation for Z^NaCl^ in the simulation model, and De was calculated. The unidirectional model simulation simulated the kinetics of NaCl diffusion during the curing process.

The simulation was performed using the COMSOL Multiphysics^®^software (version 5.5, COMSOL Inc, Stockholm, Sweden), applying the “Porous media dilute material transfer module” physical interface standard. The model’s dimensions were designed according to the actual dimensions of the experimental samples (30 mm × 30 mm × 10 mm (length × width × height)) to build a rectangular lattice geometry model for analysis. Figure 3b shows the biosolid generated by the COMSOL, with all simulations made with 3D geometry modeling with the volume subdivided into a mesh of tetrahedral finite elements consisting of the model solution domain divided by finite mesh division, generating 10,789 domain cell numbers, 8 vertex cell numbers, 120 edge cell numbers, and a minimum cell mass of 0.2608.

**Figure 4:**
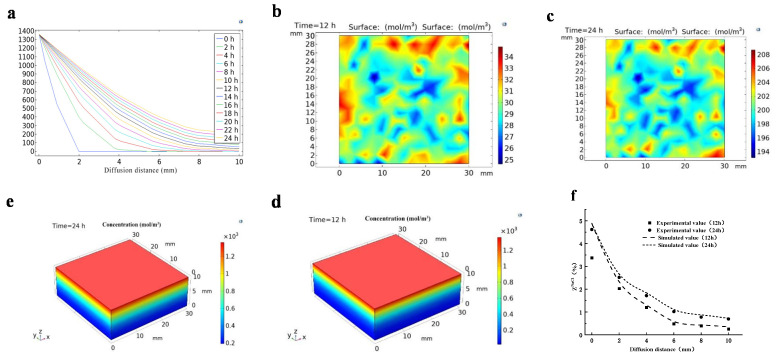
The unidimensional (**a**), 2D (**b**,**c**), 3D (**d**,**e**) simulation diagram of NaCl diffusion at different curing times with 4% xylitol, and the experimental vs. simulated values of NaCl content with 4% xylitol in pork tenderloin (**f**).

The unidimensional, 2D, and 3D simulation diagram of NaCl diffusion at different curing times with 4% (*w*/*w*) xylitol (Figure 4) showed that the NaCl content decreased when the diffusion distance of NaCl increased. Moreover, a gradual increase in the distance of NaCl diffusion into the meat was gradually observed when the curing time increased. On the other hand, when the curing time reached 12 h, NaCl could penetrate to a distance of 10 mm from the meat surface (Figure 4a). Dark blue to light pink represent low-to-high NaCl content. The 2D surface diagram (Figure 4b,c) showed that the NaCl diffusion of the pork tenderloin is uneven. In the 3D surface diagram (Figure 4d,e), different colors represent the variation in the NaCl content from dark blue to light pink, corresponding to 850–1350 mol·m^−3^. The high NaCl concentrations in the tenderloin surface might induce structural changes to the tenderloin matrix, which lead to increased NaCl diffusion [41] The results showed that most NaCl was present in the surface layer of the tenderloin (Figure 2a). During the curing, the NaCl content in the surface layer of the meat increased. However, a slow increase was detected in this content when the diffusion distance increased.

The validity of the simulation model was verified by comparing the simulated and experimental values of NaCl content. The experimental and numerical simulated values of NaCl content in the loin (Figure 4f) showed that the values of NaCl content in tenderloin have the same trend, and the NaCl content gradually decreases when the diffusion distance of NaCl increases. The results obtained from the experimental and predicted values were similar. The errors when considering 24 h of curing were 7.31% for NaCl content. This result indicates that the model has high accuracy and can be used to describe the diffusion of NaCl in curing, since they are similar to the values found by Borsato et al. [42] for the salting of quail eggs when using brine without agitation.

## 4. Conclusions

This study can prove that the numerical simulation technique could be used to investigate NaCl diffusion in cured meat products. The change in water content and NaCl content are the main reasons for the quality change of pork tenderloin during the curing process. Moreover, the change in water content is always greater than the change in NaCl. Xylitol-mediated curing can effectively reduce the NaCl content, water content, and apparent diffusion coefficient of NaCl in pork tenderloin, suggesting a promising low-salt curing method. There is a good linear relationship between the amount of xylitol added and the NaCl contents in the water phase of the pork tenderloin muscle. The concentration of xylitol has a significant impact on the mass transfer kinetic parameter K_2_ but has no significant impact on K_1_. The one-way model diffusion coefficient of NaCl was reduced from 1.29 × 10^−9^ m^2^·s^−1^ to 1.22 × 10^−9^ m^2^·s^−1^, 1.2 × 10^−9^ m^2^·s^−1^, and 1.15 × 10^−9^ m^2^·s^−1^. The results also demonstrated that the power function time-varying model had a higher correlation with experimental data (R^2^ > 0.9500). The NaCl diffusion coefficients calculated by the power function time-varying model were similar to the experimental method. The variation in NaCl distribution with position and time was solved and predicted by the COMSOL Multiphysics porous media module to analyze the diffusion process of NaCl during the curing process more intuitively. At the beginning of curing, the NaCl content in the pork tenderloin surface increased rapidly, increasing from 0 mol·m^−3^ to 1350 mol·m^−3^ from 0 to 12 h. Furthermore, the NaCl content increased slowly when the diffusion distance was enhanced. The NaCl penetrated to a position 10 mm from the meat surface, observed after 12 h. Based on the simulation results, a more accurate understanding of the diffusion mechanism was obtained. The model’s predicted values were validated against the experimental values, indicating that the model is reasonable. Overall, this research work suggests that using COMSOL Multiphysics to describe the spatial distribution of NaCl is a very economical and fast method, and no expensive instruments were required to achieve these results. Therefore, this study provides a new idea for achieving intelligent production in the food industry. In future studies, it is also necessary to develop other studies to evaluate the physicochemical properties during dry-ripening and the characterization of final product quality (oxidative status, physicochemical properties, and sensory properties).

## Figures and Tables

**Table 1:** Kinetic parameters and fitting correlation coefficients of pork tenderloin cured in different xylitol concentrations.

**a. Kinetic Parameters of Pork Tenderloin Cured in Different Xylitol Concentrations**				
**Addition of Xylitol/(*w*/*w*)**	**De/(10^−9^ m^2^ s^−1^)**	**K**		**R2**
0%	2.58 ± 0.12	−0.1060		0.9569
4%	2.25 ± 0.10	−0.1272		0.9327
8%	2.20 ± 0.03	−0.1697		0.9065
12%	2.35 ± 0.08	−0.2088		0.9001
**b. Kinetic Parameters for Δ$M_{t}^{0}$, Δ$M_{t}^{w}$, and Δ$M_{t}^{NaCl}$ and Fitting Correlation Coefficients**				
**Variables**	**Addition of xylitol/(*w*/*w*)**	**K1**	**K2**	**R2**
Δ$M_{t}^{0}$	0%	0.94972	0.066870	0.9464
	4%	0.95896	0.056627	0.9533
	8%	0.98067	0.047707	0.9775
	12%	0.96797	0.032758	0.9160
Δ$M_{t}^{w}$	0%	0.95202	0.060436	0.9403
	4%	0.97873	0.047178	0.9669
	8%	0.98555	0.036387	0.9650
	12%	0.97978	0.029523	0.9500
Δ$M_{t}^{NaCl}$	0%	0.99072	0.010715	0.9189
	4%	0.99204	0.009633	0.9246
	8%	0.99316	0.008951	0.9302
	12%	0.99345	0.008060	0.9232

**Table 2:** Equations of three prediction models and calculated De values.

Predictive Model	Addition of Xylitol (*w*/*w*)	Equation	R^2^	De (10^−9^ m^2^·s^−1^)
linear function time-varying model	0%	Z^NaCl^ = 0.0014143t + 0.0294540	0.6238	1.57
	4%	Z^NaCl^ = 0.0013564t + 0.028745	0.6117	1.47
	8%	Z^NaCl^ = 0.0013992t + 0.0280392	0.6313	1.5
	12%	Z^NaCl^ = 0.0013282t + 0.0262829	0.6461	1.36
Power function time-varying model	0%	Z^NaCl^ = 0.026587t^0.212376^ + 0.005467	0.98558	1.29
	4%	Z^NaCl^ = 0.025606t^0.212376^ + 0.005471	0.981743	1.21
	8%	Z^NaCl^ = 0.024312t^0.212376^ + 0.005456	0.978139	1.14
	12%	Z^NaCl^ = 0.022290t^0.212376^ + 0.005470	0.981368	1.01
Exponential function time-varying model	0%	Z^NaCl^ = 0.053269(1 − e^−0.408018t^)	0.952283	1.21
	4%	Z^NaCl^ = 0.051846(1 − e^−0.3930302t^)	0.962545	1.16
	8%	Z^NaCl^ = 0.0523298(1 − e^−0.3473287t^)	0.968102	1.23
	12%	Z^NaCl^ = 0.04943(1 − e^−0.33598t^)	0.960972	1.14

## Data Availability

Data are contained within the article.
